# Urban Residents’ Acceptance Intention to Use Recycled Stormwater—An Examination of Values, Altruism, Social and Cultural Norms, and Perceived Health Risks

**DOI:** 10.3390/ijerph19052825

**Published:** 2022-02-28

**Authors:** Shufen GUO, Zhifang Wu, Ludi Wen

**Affiliations:** 1 Cooperative Innovation Center for Transition of Resource-Based Economies, Shanxi University of Finance and Economics, Taiyuan 030006, China; sdguosf@163.com; 2 UniSA Business, University of South Australia, Adelaide, SA 5001, Australia; 3 School of Business Administration, Shanxi University of Finance and Economics, Taiyuan 030006, China; wenludi950425@163.com

**Keywords:** stormwater reuse, altruism, social and cultural norms, perceived health risks, public acceptance

## Abstract

Public acceptance is the basic premise for the implementation of stormwater reuse projects anywhere in the world. Based on the theory of planned behaviour, this study constructed a hypothesized model of urban residents’ intention to use recycled stormwater for non-potable residential purposes. Having received 669 valid questionnaires from urban residents in Taiyuan City, a Structural Equation Model was used to analyze their acceptance intention to use recycled stormwater. Results of the study showed that the degree of human contact with recycled stormwater influenced respondents’ acceptance intention to use it for that purpose, which is consistent with previous studies. The impact of factors, including valuation of stormwater, emotions, perceived health risks, or trust in government, on respondents’ acceptance intention to use recycled stormwater was found to be not significant, which adds to the inconsistent literature. The unique contributions of the study to literature include that altruism and social and cultural norms were found to have significantly positive impacts on residents’ acceptance intention to use the water, while social and cultural norms demonstrated a more significant impact. This finding is perceived to relate to the collectivism of Chinese culture; however, to what extent the relation could be requires further research to verify. The study also makes contributions to methodology by using social networking (WeChat Moments) to collect data in social science studies.

## 1. Introduction

The extensive execution of environmental policies and strategies around the world has a significant impact on water and energy usage and waste management [1]. Researchers argued that such policies stress on environmental communication [2], encourage purchasing energy-saving and reusable materials, and promote recycling and resource conservation [3]. Sakshi et al. [4] in their recent study, further highlighted the importance of such resource and energy conservation in improving environmental performance and financial performance.

Stormwater is defined as surface runoff in urban areas such as roads, sidewalks, roofs, parking lots, and open space [5]. Recycled stormwater is increasingly being considered as one of the complementary or alternative sources of water to increase freshwater supplies, promote resource conservation, and meet the growing demand for humans [6].

Stormwater reuse refers to harvesting stormwater through specific pipes during the rainy season, and then treating the harvested stormwater to a quality that meets the required standards for specific uses. In addition to augmenting freshwater supplies, recycling urban stormwater can reduce urban flooding, reduce urban sewage treatment load, and alleviate water shortage. Compared with desalination, trans-basin water transfer or sewage treatment reuse, stormwater reuse has the unique advantages of less pollution and easier treatment, and has good environmental, economic, and social benefits [7]. 

In recent years, stormwater reuse has gradually gained the attention of both policymaking and the public. Some countries and regions have treated stormwater for residential uses which have low quality requirements and achieved good economic and ecological benefits [6]. For example, the Australian Managed Aquifer Recharge (MAR) system harvests stormwater during the rainy season, injects it into underground aquifers for filtration and storage, then extracts and treats the water during the dry season for residential uses [8]. The range of potential uses of recycled stormwater have expanded due to the advances in stormwater treatment technology, but public acceptance remains to be the promotion bottlenecks faced by stormwater reuse projects [5,9,10,11]. 

This study constructed a hypothesized model of urban residents’ intention to use recycled stormwater for non-potable residential purposes. The data was collected with Taiyuan City residents through a questionnaire that was posted via a social media channel WeChat. Within a week, 669 valid questionnaires were received. A Structural Equation Model was used to analyze respondents’ acceptance intention to use recycled stormwater. Based on Ajzen’s theory of planned behaviour [12], the study incorporated variables such as valuation of stormwater, trust in government, altruism, social and cultural norms (which relate to Chinese cultural values), emotions, and perceived health risks. It provided an understanding of urban residents’ intentions to accept using recycled stormwater for various residential purposes in a highly collectivistic country—China, an emerging country in Asia which is largely different from western/developed countries in many aspects including population size, urban dwelling style, and social and cultural norms. The study aimed to address research questions, including:

1. How would a highly collectivistic community perceive using recycled stormwater for residential purposes?
2. What are the potential factors that influence respondents’ decision making regarding accepting using the recycled stormwater for residential purposes?
3. To what extent does each of the influencing factors affect respondents’ acceptance intentions?

The rapid economic and urban development of Taiyuan, like many Chinese cities, requires massive water resources consumption [13]. However, the city faces the most severe water scarcity problems. Taiyuan’s water resources per capita is 173 m^3^, which is less than 1/10 of the national per capita level. Water scarcity has been seriously restricting the sustainable development of the city [14]. On the other hand, stormwater is discharged directly into sewage, with little being recycled.

To implement the 2030 Agenda for Sustainable Development [15], China has established the first few innovation demonstration zones, and Taiyuan City, Shanxi Province, is one of the first three national sustainable development agenda innovation demonstration areas in China [16]. The city has committed to augment their water supply by using non-conventional water sources, and to provide an effective model for the implementation of the 2030 sustainable development agenda for the nation. In this context, Taiyuan Municipal Government has enacted the Development Plan for the Innovation Demonstration Zone of the Sustainable Development Agenda of Taiyuan City (2017–2030). The plan outlines the determination of Taiyuan government to solve the problem of urban water shortage, with one of the important proposals being “stormwater reuse”. 

While the required technology to maintain water safety is readily available, public acceptance is the basic premise for the implementation of stormwater reuse projects anywhere in the world [17]. The social science study on public acceptance for using non-conventional waters, including recycled wastewater and recycled stormwater, originated early in this century. Smith and other scholars reviewed existing research in this field and found an important gap, which is “a relative lack of longer-term longitudinal studies that document whether/how support for reuse schemes can shift over time” [18] (p. 44). While we agree with Smith et al.’s statement, we found another important research gap in this area, which is that such studies were rarely conducted in developing or emerging countries. So far, most of the existing research in the area is concentrated in the United States, Israel, Singapore, or Australia, and rarely in developing countries [5,6,9,10,11,19,20,21,22,23]. Very limited research has been carried out in a large emerging country like China in which population is very different in dimensions such as size, values, social and cultural norms, or dwelling types compared to developed western countries. 

The limited research on stormwater reuse in China has been focusing on engineering technology or investment return estimates. This research lacks extensive research on public acceptance for using the recycled stormwater and the potential factors influencing their urban residents’ decision-making. What urban residents—the main and end user group—perceive in using the recycled stormwater for residential purposes should be the basic premise for a successful implementation of stormwater reuse projects. The present study aims to address this research gap.

### 1.1. Study Problem

Culture plays a vital role in shaping people’s intentions and behaviour [24]. Research on the cultural dimension such as collectivism and individualism has provided interesting insights for us to understand why people make environmental decisions [25]. Culture is identified as crucial to understand success of, or barriers to, the implementation of new management strategies [26]. It also helps to exchange experience between developed and developing countries [26]. Researchers have stressed the need for studies in the context of developing or emerging countries because cultural values orientations in these countries are different from those in developed countries [27]. Specifically, cultural values can be a fundamental characteristic that impacts the pro-environmental consumption behaviour of consumers. For example, research evidence suggests that citizens in Asian countries are increasingly becoming concerned about alarming environmental problems and policymaking in many Asian countries are giving more consideration to long-term sustainable developments, including environmental protection and resource reuse [28,29,30,31]. 

The present study therefore included variables such as values, altruism, social and cultural norms, and trust in government that relate to Chinese cultural values, based on the current literature on cultural values and behaviour, and with an effort towards understanding the relationships in the Chinese context of intervening environmental attitudes and behavioural intentions. The study problem is that in a highly collectivistic society such as China, do values, emotions, perceived health risks, altruism, social and cultural norms, or trust in government influence urban residents’ acceptance intention to use recycled stormwater for residential purposes?

### 1.2. Hypotheses

It is generally accepted in the field of environmental behaviour that behavioural intention acts as an intermediary between all psychosocial variables (including attitudes, norms, senses of control, values, etc.) and environmental behaviour [23]. Urban residents’ intention to accept stormwater reuse is a study of intention, which is generally considered to reflect the size of the individual’s likelihood of carrying out a particular act [12]. 

Studies on the acceptance intention to use recycled stormwater, which were mostly conducted in Australia, have found that residents’ intention to use the water is influenced by psychological, social, and policy factors. Based on the theory of planned behaviour [12], we draw on existing studies [6,7,8,9,10,11,32,33] to test the impact of (1) psychological factors, including value judgment, emotion, perceived health risks, and altruism; (2) social factors such as social and cultural norms; and (3) policy factors such as trust in government, on the intention of Taiyuan’s urban residents to accept using recycled stormwater for residential purposes. 

Most urban residents have a positive view of the value of stormwater, arguing that collecting stormwater is a form of “saving” and that the flow of stormwater into sewage is wasteful [5]. However, the impact of the stormwater valuation on their acceptance intention to use recycled stormwater remains unclear. In Wu et al.’s study [6] in Australia, more than 80 percent of respondents considered stormwater to be a valuable resource, and stormwater reuse was critical to addressing water scarcity, but their value judgment about stormwater did not impact their attitude and acceptance intention to use recycled stormwater. Some researchers assumed that the insignificant relation might be due to residents’ perception of water quality or their personal experience, which may lead some users to not intend to use water given a concern of health safety [34]. Therefore, residents’ value judgments about stormwater may or may not impact their intention to accept stormwater reuse. 

It became our research interest as to whether residents’ valuation of stormwater in a different cultural context shapes their acceptance intention towards using recycled stormwater. Man-to-nature orientation value, which originates from Taoism, is considered to be one of the Chinese traditional cultural values [35]. It is a cultural value leaning toward environmental friendliness [35]. Researchers found that man-to-nature orientation value could significantly affect Chinese consumers’ pro-environmental purchase intention [36,37]. Wang and Wu discovered in particular that the man-to-nature values played fundamental roles in appealing to household water saving behaviour [31]. Combing the connotation of man-to-nature orientation value and related studies, we can deduce that Chinese residents have positive views of the value of stormwater, and, accordingly, the value has a positive facilitative impact on their pro-environmental behaviour intention; therefore, we propose:

**Hypothesis** **1.** *In a highly collectivistic community, the more valuable urban residents consider stormwater is, the more willing they are to use recycled stormwater; i.e., valuation of stormwater has a positive impact on intention to accept using recycled stormwater.*

Emotion is one of the most controversial topics in psychological research, and emotions are often considered irrational and difficult to measure accurately [37]. In the studies on water recycling for residential uses, the discussion about emotions focused mostly on negative emotions [32]. Empirical studies in Australia have shown that negative emotions about stormwater reuse (e.g., “yuck factor” or “feeling sick”), but have no direct negative impact on their intention to use the water [6,32]. Discovering whether emotional factors impact residents’ behavioural intention to use recycled stormwater in a different social and cultural context was a worthwhile investigation. We then propose:

**Hypothesis** **2.** *In a highly collectivistic community, the higher the urban residents’ aversion to the use of recycled stormwater, the less willing they are to use it; i.e., negative emotions have a negative impact on intention to accept using recycled stormwater.*

Perceived health risks are an important factor in environmental behavioural research, and some scholars believe that perceived health risks can indirectly affect residents’ acceptance intention to use the recycled stormwater [6,19]. Their findings are converse to an earlier study by the Australian Federal Scientific and Industrial Research Organization (CSIRO) which suggests that health risks do not significantly affect people’s decisions to accept or reject stormwater reuse [38]. These studies were conducted in Australia. The present study is keen to test the influence of perceived health risks on residents’ acceptance intention towards using recycled stormwater in a different social and cultural context, provided the water meets the corresponding water quality standards in the city. So, we therefore propose:

**Hypothesis** **3.** *In a highly collectivistic community, when urban residents consider that the use of recycled stormwater threatens the health safety of themselves, their family, or their community, they are unlikely to use the water; i.e., perceived health risks have a negative impact on intention to accept using recycled stormwater.*

Trust in government refers to the normative belief that government operates in accordance with people’s expectations of how a government should operate [39]. Some scholars refer to the role of government trust in political support as a trust heuristic; i.e., the more people trust the government, the more likely they are to support government policies or other calls [40]. Trust in government is an important determining factor in public satisfaction with water recycling programs [41], as well as in public acceptance of a variety of water sources, including stormwater and desalination [10]. Studies on recycling stormwater for potable or non-potable uses have shown that trust in government authorities is the main indicator of residents’ acceptance of stormwater for drinking purposes [5], while the effect on their intention to use the recycled stormwater could be indirect [42]. 

Researchers believe that most collectivists are willing to prioritize group goals over their own personal ones; hence, they are more likely to act in an environment-friendly manner because it is good for the group [43,44]; and hence, they are often more concerned for the public good. Many feel obliged to protect the environment so that their groups can enjoy prosperity [23]. They are therefore more likely to demonstrate cooperative behaviour upon government calls associated with public good consumption [43,45]. Accordingly, we therefore propose:

**Hypothesis** **4.** *In a highly collectivistic community, residents are more likely to trust their government; the more residents trust in their government, the more willing they are to use recycled stormwater; i.e., the degree of trust in government has a positive impact on intention to accept using recycled stormwater.*

Altruism is the antithesis of self-interest and altruistic behaviour is the act of voluntarily helping others without expecting an external reward [46]. Altruistic people tend to help others to ensure that benefits are passed on to future generations [47]. People feel a sense of duty and responsibility toward their community and next generations [48,49]. Altruism has an interpretation similar to Nowell and Boyd’s concept of community responsibility, which is “a feeling of personal responsibility for the individual and collective well-being of a community of people” [50] (p. 231).

In a study of pro-environmental consumption, altruistic consumers were found paying more attention to the ecological benefits of their behaviour than to their own benefits [51]. Panda et al.’s study [52] also found that altruism has a significant positive impact on consumers’ intention to buy pro-environmental products. In a cultural context, researchers argued that collectivistic cultures are more strongly tied to altruistic motivations [53]. We accordingly propose:

**Hypothesis** **5.** *In a highly collectivistic community, the higher the altruistic tendency of urban residents, the more they would consider the social and ecological benefits of stormwater recycling, which would tend to an acceptance intention towards using recycled stormwater; i.e., altruism has a positive impact on intention to accept using recycled stormwater.*

Social and cultural norms reflect what people do in different situations and what they think others should do [29,30], which is influenced by many factors, such as values, cultural practices, social traditions, and so on. It plays a vital role in shaping individuals’ intentions and behaviour [24]. If personal behaviour deviates from the social and cultural norms of the community they belong to, psychological and social pressures such as guilt can arise. In environmental behaviour studies, social and cultural norms of recycling behaviour affect people’s recycling and other sustainable behaviours [54]. 

The influence of social and cultural norms on pro-environmental purchase behaviour has been studied by many researchers [29,30,55,56,57]. These studies were conducted in both developed and developing countries. Their findings are consistent with previous research that claims normative social influence is especially helpful in explaining consumer behaviours in collectivist cultures such as China, India, and Iran [29,30,55,56].

Due to the influence of Confucius culture, a group-oriented culture, Chinese people have a cultural tendency toward collectivism, which encourages people to comply with social norms rather than personal goals [58]; the consumers under the influence of such a culture are therefore largely influenced by their family members, neighbors, friends, colleagues, and even society as a whole [59]. 

To date, we have found a small number of studies in Australia [6,11] which studied the impact of social norms on residents’ intention to accept stormwater reuse, and their findings are inconsistent. To the best of our knowledge, no such study has been conducted in developing or emerging countries. Thus, the present study aims to bridge this literature gap to offer an interesting avenue of insight which may help us to understand how people make decisions in those regions. We then propose:

**Hypothesis** **6.** *In a highly collectivistic community, when urban residents perceive that people around them would be or are using recycled stormwater, if they do not use it, they would consider that they are not conforming to social and cultural norms or even feel guilty. The more people around an individual use recycled stormwater, the more likely the individual would use recycled stormwater; i.e., social and cultural norms have a positive impact on intention to accept using recycled stormwater.*

### 1.3. Conceptual Model Construction

**Figure 1:**
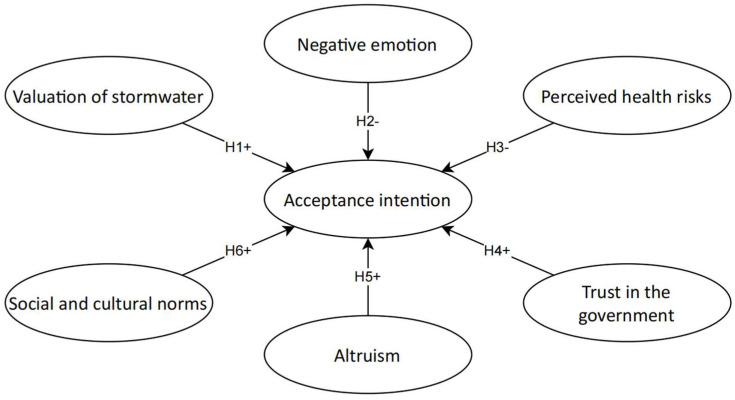
Conceptual model of public acceptance intention towards using recycled stormwater. Note: + positive impact; − negative effect.

Based on Ajzen’s theory of planned behaviour [12], the study constructed a conceptual model to test the influencing factors’ impact on urban residents’ acceptance intention towards using recycled stormwater for residential purposes (Figure 1). We modified the framework of the theory of planned behaviour by including culture-relating variables (valuation of stormwater, altruism, trust in government, social and cultural norms) and personal perception variables (negative emotions and perceived health risks). The study adds to the existing sustainability literature by identifying central pro-environmental cultural value orientations: man-to-nature, collectivism, altruism, and future orientation, integrating them into the underlying model of planned behaviour.

The use of recycled stormwater is usually divided into potable and non-potable uses [11], and non-potable uses are further subdivided into more specific uses by researchers depending on the degree of close contact with body [33]. Studies have shown that residents’ intention to use recycled water is related to the intended uses of the water; that is, the higher the degree of close contact with human body, the lower the willingness people present to use the water [14,29,56]. Considering the low public acceptance for using recycled stormwater for potable purposes, which often involves high treatment cost and safety standards, we followed the existing research [29], and only tested using recycled stormwater for non-potable residential uses, including:

(1) personal washing
(2) clothes washing
(3) pet washing
(4) indoor plant watering
(5) car washing
(6) toilet flushing
(7) outdoor green irrigation
(8) road spraying

The intended uses were modified by taking into consideration the residential styles of Taiyuan residents (city apartment dwellers who love to have indoor plants). Accordingly, the model in Figure 1 gets tested 8 times for the 8 intended uses to explore how each of the 6 influencing factors impacts urban residents’ intention to accept using recycled stormwater for each use in a highly collectivistic community.

## 2. Materials and Methods

The study used survey techniques to capture residents’ responses to the research topic. Surveying is the most used technique to collect data in the field of research. It has great advantages in reaching out to a relatively large sample, but it meanwhile has disadvantages in exploring respondents’ thoughts deeply. Hence, the present study planned to conduct a focus group discussion with respondents to gain further information about their acceptance intention to use recycled stormwater and the potential factors that affect their decision-making.

The survey items mainly drew on existing research on environmental behaviour measurement [6,19,60,61]. The questionnaire contained 31 questions in three parts. The first part was to gain participants’ demographic profiles to reflect the representativeness of the sample. The second part included a few “warm-up” questions to introduce important concepts, given residents’ limited knowledge of non-conventional water sources or stormwater reuse projects. The third part used a 5-point Likert scale to measure urban residents’ acceptance intention to use recycled stormwater and its possible influencing factors, which included valuation of stormwater, negative emotions, perceived health risks, trust in government, altruism, and social and cultural norms. 

We piloted the survey offline and online. The offline survey (hard copies) was piloted with Xinghewan Community and Eryingpan Community in Taiyuan (Taiyuan has 6 large residential communities, including Xiaodian, Yingze, Wanbailin, Xinghualing, Jiancaoping, Jinyuan), of which the Xinghewan Community currently has some small-scale stormwater reuse facilities, while the Eryingpan Community has not yet built stormwater reuse facilities. The offline survey was collected with the help of community property management offices (which manage communal matters for the community) and the street offices (which is part of Local Government Authorities). The online survey was mainly collected through WeChat Moments (a Chinese social network application like Facebook) in which the survey QR code for online filling was shared with Taiyuan residents except the residents who live in Xinghewan or Eryingpan Community. 

A total of 209 completed questionnaires were collected through the pilot survey. The pilot process suggested that the offline questionnaires were more difficult to reach respondents, and the sample was more likely biased. We were only allowed to distribute questionnaires in the public leisure areas of the community, where the population are mostly elderly. It was determined that the final survey was to be collected online using WeChat Moments. In total, 669 valid responses were received for the final survey, indicating an effective response rate of 83.5%. The study makes contributions to methodology by using social networking (WeChat Moments) to collect data in social science studies. 

Prior to administering the survey, the survey items and responses were back translated to ensure accuracy of translation. Missing values were less than 1% for each item and were treated using a regression imputation on AMOS 26, SPSS (IBM, Chicago, IL, USA) The measurement model test included confidence and effectiveness test. Valuation of stormwater, negative emotion, perceived health risk, trust in government, altruism, and social and cultural norms were set as exogenous subvariance variables, and the acceptance intention was set as an endogenous variable. The standardized Cronbach’s α value of 0.944 for all measurements in the questionnaire indicated that the overall confidence of the questionnaire was high. The Cronbach’s α of the seven latent variables were all greater than 0.7, indicating that the corresponding observational variables had a good internal consistency. AMOS 26 was further used for validation factor analysis, and the convergence of the model was tested by construct reliability (CR) and average variance extracted (AVE). The CRs of each of latent variables were greater than 0.8, and the AVEs were greater than 0.5. This indicated that the convergence of the measurement model passed the tests [62], as detailed in Table 1.

Given a satisfactory level of reliability and validity of the measurement model, this study proceeded to test the structural model. The structural equation model was estimated by Maximum Likelihood Estimation method, and the fit indices of the structural model were all within the fit criteria or critical value range (Table 2) in 8 tests, indicating that the structural model had a good fit, and the theoretical model fit well with the actual data [62].

## 3. Results and Discussion

The demographic profile of respondents (Table 3) indicated a good representation compared with the statistical data published by the Statistical Bureau of Taiyuan City, except age and gender. People aged over 50 years had a smaller percentage, which might be due to the online data collection method assuming elderly people are less likely to be available online. Women presented a higher participation percentage, which could be explained by Lee’s finding that women had higher environmental concern and were more environmentally responsible compared to men, leading to a higher percentage of participation in such surveys [63].

Results of the survey showed that the urban residents in Taiyuan city had a strong sense of water conservation. Regarding how to solve water shortage problem in Taiyuan City, saving water (reduce water consumption) was ranked as the top one measure, which was followed by stormwater reuse, sewage treatment, cross-basin water transfer, government water restrictions, and groundwater extraction. Stormwater reuse was ranked as the second, suggesting a positive attitude of urban residents towards stormwater reuse. The positive attitudes were further demonstrated by respondents’ valuation of stormwater. Overall, respondents considered stormwater has great value, agreeing that “the blind discharge of stormwater results in the waste of stormwater resources” (mean = 4.141, SD = 0.829), “stormwater is a valuable natural resource, and its recycling should be greatly promoted (mean = 4.348, SD = 0.760)”, and “reasonable reuse of stormwater can alleviate the problem of urban flooding when the rain is abundant” (mean = 4.306, SD = 0.742). While the findings are consistent with those of Wu et al.’s study in Australia [6], Chinese residents expressed a slightly higher rating on same statements in terms of stormwater valuation. We assume this is due to the man-to-nature characteristic of Chinese culture, which was originated from Taoism. 

Respondents also demonstrated a high level of trust in government, agreeing that “If the stormwater reuse program is implemented, I trust the information provided by the government regarding the safety of the water” (mean = 4.133, SD = 0.816), and “If the stormwater reuse program is implemented, I trust government authorities to ensure that water quality is safe and meets health and hygiene standards” (mean = 4.048, SD = 0.876). As discussed previously, in general, we can expect that in collective societies residents are willing to prioritize group goals over their own personal that will more easily lead to an acceptance of shared goals [26], and more likely to demonstrate trust in or cooperative behaviour upon government calls associated with public goods [40]. Hence, it is not surprising that Chinese residents are more likely to trust their government in relation to managing an alternative water supply that is believed as good for their group [43,44,45]. 

**Figure 2:**
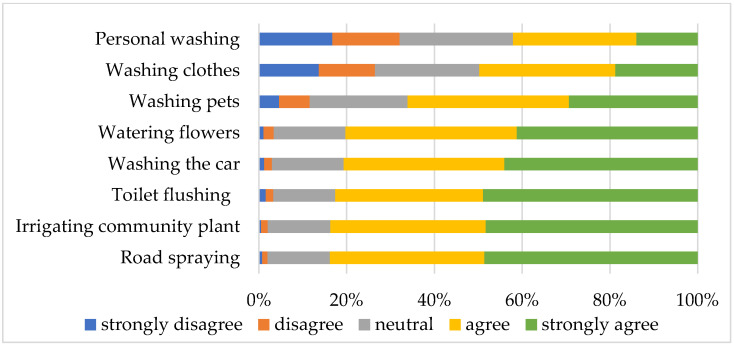
Urban residents’ acceptance intention to use recycled stormwater for various residential uses.

The results of exploring urban residents’ intention to accept using recycled stormwater for the 8 residential uses are illustrated in Figure 2. It was found that residents had a higher acceptance intention for uses that have less contact with the human body, such as indoor plant watering, car washing, toilet flushing, outdoor green irrigation, or road spraying. Their intention to use became lower for uses that have a higher degree of contact with human body, such as personal washing, clothes washing, or pet washing. The finding is consistent with that of Dolnicar and Grun’s research [19] on recycled wastewater, and of Wu at al. [33] and Mankad et al.’s research [11] on stormwater reuse, suggesting that the degree of human contact can significantly affect residents’ intention to use recycled stormwater. It is believed that the finding has important implications for the promotion and implementation of stormwater reuse projects. It would probably be wise or more feasible to start with supplying recycled stormwater for uses that have less contact with the human body, which often involves lower treatment cost and less advanced technological application. 

The study of Dolnicar and Grun [19], Wu et al. [33] or Mankad et al. [11] were all conducted in Australia. The country is largely different from China in terms of population, urban dwelling style, or social and cultural norms. The consistent findings in two different contexts suggest that such differences do not have significant influence on their residents’ acceptance intention to use recycled stormwater for purposes that have close or less close contact with human bodies. 

The acceptance or rejection of hypotheses are illustrated in Table 4. Hypothesis 1 was rejected in the tests for all intended uses except test 4 (indoor plant watering). The finding is consistent with that of Wu et al.’s study [6] in Australia that residents’ valuation of stormwater did not affect their intention to accept using recycled stormwater. Whether the insignificant influence was due to residents’ perception of water quality or their personal experience [34], requires further research such as a focus group study or in-depth interviews (under planned). The significant result of test 4 (at *p* < 0.01 level) suggests that the more valuable residents consider stormwater is, the less willing they are to use recycled stormwater to water their indoor plants, was a contrary to expectations also requiring further explorations. 

Hypothesis 2 was rejected in the tests for all intended uses. Negative emotions were found not significantly impacting residents’ intention to accept using recycled stormwater for all intended uses. This finding supports the empirical studies in Australia which found that negative emotions about stormwater reuse (e.g., “yuck factor” or “feeling sick”) had no direct negative effect on their intention to use the water [6,32]. However, researchers need to bear in mind that emotions are often considered irrational and difficult to measure accurately [37]. Hence, it remains a controversial factor in present research area.

Hypothesis 3 was rejected in the tests for all intended uses. In the context of recycled stormwater meeting the necessary water quality standards in Taiyuan City, perceived health risks were found not significantly impacting residents’ intention to accept using the water. The finding supports the earlier study by the Australian Federal Scientific and Industrial Research Organization (CSIRO) [38]; while is inconsistent with that of Dolnicar and Grunand [19] or Wu et al. [6] that found perceived health risks can affect residents’ intention to use the recycled stormwater. 

Hypothesis 4 was also rejected in the tests for all intended uses, indicating that residents’ trust in government did not have significant impacts on their intention to accept using recycled stormwater. Though respondents demonstrated a high level of trust in government to ensure that water quality is safe and meets health and hygiene standards, their trust did not demonstrate significantly positive impacts on their intention to use the water. The finding is consistent with the finding of Wu et al. [6], but may not support the study of Mankad et al. or Ross et al., who found trust in government authorities had effects on intention to use recycled stormwater though indirectly [5,42]. 

However, Hypothesis 5 was accepted at a significant level (*p* < 0.001) in the tests for all intended uses. The higher the residents’ altruism tendencies were, the residents were more willing to use recycled stormwater for the intended uses. Altruism had a significantly positive impact on residents’ intention to accept using recycled stormwater, with a medium effect size (ranging between 16–29%). The finding supports the statements of Gronlünd et al. [53], Steg et al. [51] and Panda et al. [52] that altruism has a significant positive impact on consumers’ intention to buy pro-environmental products because altruistic consumers are paying more attention to the ecological benefits of their behaviour than to their own benefit. In a cultural context, collectivistic cultures (e.g., Chinese cultures) are strongly tied to altruistic motivations [51,52,53]. Chinese residents tend to help others and often feel a sense of duty and responsibility toward their community and next generations [29,30,59]. 

Hypothesis 6 was also accepted at a significant level (*p* < 0.001) in the tests for all intended uses, suggesting that social and cultural norms had a significantly positive impact on residents’ intention to accept using recycled stormwater. The effect size was high (ranging between 60–70%). The finding is consistent with previous empirical studies [29,30,55,56,57] and supports their argument that normative social influence is especially helpful in explaining consumer behaviours in collectivist cultures such as China, India, or Iran [29,30,55,56]. However, to what extent this finding could be attributed to the collectivism of Chinese culture, which is the typical and traditional cultural value in China and the opposite to Western Culture, requires further research to verify.

## 4. Conclusions and Managerial Implications

Researchers advocate that it is crucial to understand barriers to the implementation of technologies and new management strategies in pro-environmental areas [26,64]. This reflects an emphasis shift from focusing on “hard” technology to a “soft” path in water management encouraging end-users’ participation to ensure meeting end users’ needs [26,65]. They also argue that a successful exchange of experience between developed and developing countries in such areas is important to global well-being and sustainability [26,64]. 

Previous research on exploring residents’ (end users) acceptance for using recycled stormwater were mostly conducted in developed countries such as Australia. Few studies were conducted in developing or emerging countries such as China, which is largely different from western/developed countries in many aspects. Further, amongst the limited research on stormwater reuse in China, most of them focused on engineering technology or investment return estimates, and little studied public acceptance for using recycled stormwater, not mentioning of what potentially influence residents’ intention to use the water. This study contributes to the body of literature in the field by proposing and testing an integrated model which incorporates six variables, namely, valuation of stormwater, emotions, perceived health risks, trust in government, altruism, social and cultural norms. Four of the six variables including valuation of stormwater, trust in government, altruism, social and cultural norms relate to Chinese cultural values. The findings of our study offered theoretical implications and provide answers to the following research questions: (a) how a highly collectivistic community perceive using recycled stormwater for residential purposes; (b) what affects their acceptance intention to use recycled stormwater for residential purposes; and (c) to which extent each of the influencing factors affects their acceptance intention.

Results of the study showed that the degree of human contact with recycled stormwater influenced respondents’ acceptance intention to use for that purpose, which is consistent with previous studies. The impact of factors, including valuation of stormwater, emotions, perceived health risks, or trust in government, on respondents’ acceptance intention to use recycled stormwater was found not significant, which adds to the inconsistent literature. The unique contributions of the study to literature include that altruism, social, and cultural norms were found having significantly positive impacts on residents’ acceptance intention to use the water, while social and cultural norms demonstrated a more significant impact. This finding is perceived to relate to the collectivism of Chinese culture; however, to what extent the relation could be requires further research to verify. 

While the study made contributions to literature through presenting new findings in the area, it at the same time provided managerial implications for managers or practitioners regarding a more feasible or more cost-effective implementation of urban stormwater reuse projects. 

Overall, this study found that the acceptance of urban residents in Taiyuan for using recycled stormwater was high. It is evidenced that residents’ acceptance for using recycled stormwater for uses that have less close contact with the human body is higher than using it for uses that have close contact with human body. It would be more feasible to start with supplying recycled stormwater for uses that have less close contact with the human body, which often involves in lower treatment cost and less advanced technological application. 

The positive effects of altruism, social and cultural norms indicate a great potential of effective promotion of stormwater reuse, which could be delivered via the wide use of social media such as Weibo or WeChat in China. When part of the community starts using recycled stormwater, more residents would follow on. In other words, if stormwater reuse projects could be successfully launched in a small number of communities, it could be rolled out on a larger scale. 

China’s rapid economic and population growth have created some significant environmental crises [66]. Pro-environmental consumption behaviours are largely encouraged in order to achieve the sustainability of a healthy population and economy. Understanding the unique values of Chinese residents can provide valuable insights for developing social policy and marketing strategies that encourage pro-environmental consumption behaviours. Governments or businesses should not only focus on introducing pro-environmental products and their associated benefits, but also address the social and cultural context in which the products are consumed and disposed of, as these factors will also influence both the cognitive and behavioural aspects of pro-environmental behaviour. 

Stormwater management is the responsibility of state, municipal, and local governments. The evidence received about the roles that different level of government and water utilities perform in stormwater management could be viewed in regions where stormwater recycling and management has a relatively long history, such as the South Australia, Australia. South Australia emphasizes the leadership role of councils (local governments) to play in stormwater management and the importance of their leadership through formal strategic planning and policy development [67]. The State is leading the world in stormwater harvest and reuse [6,33]. Therefore, its experience and success could be followed by the managers and practitioners in the regions or countries such as China, where a stormwater reuse program is to be planned or implemented. 

## 5. Research Limitation and Further Research Opportunities

Studies on urban residents’ intention to accept alternative water sources have shown that the cost of water could also be an important factor in residents’ water use decisions [19]. In general, the cost of treating alternative water is higher than the cost of treating tap water, but people want to pay a lower fee for alternative waters [32]. The present study did not include the pricing of recycled stormwater into investigation due to the unavailability of required data, which was considered to be a research limitation.

Altruism and social and cultural norms, which are believed relate to the collectivism of Chinese cultural values, were found having significantly positive impacts on residents’ acceptance intention to use recycled stormwater for residential purposes. However, the question as to what extent the relation may be requires further research. A well-designed focus group discussion or in-depth interview would be appropriate. 

## Figures and Tables

**Table 1:** Reliability and validity of the measurement model.

		Test 1	Test 2	Test 3	Test 4	Test 5	Test 6	Test 7	Test 8
Valuation of stormwater	α	0.851	0.851	0.851	0.851	0.851	0.851	0.851	0.851
	CR	0.854	0.853	0.853	0.854	0.854	0.854	0.855	0.854
	AVE	0.662	0.661	0.661	0.662	0.662	0.662	0.663	0.663
Negative emotion	α	0.846	0.871	0.856	0.853	0.884	0.875	0.873	0.884
	CR	0.849	0.872	0.857	0.855	0.885	0.876	0.874	0.885
	AVE	0.737	0.773	0.749	0.747	0.793	0.779	0.776	0.793
Perceived health risks	α	0.810	0.816	0.820	0.797	0.832	0.830	0.836	0.851
	CR	0.815	0.823	0.825	0.803	0.835	0.832	0.838	0.853
	AVE	0.689	0.701	0.702	0.672	0.718	0.712	0.722	0.743
Trust in government	α	0.866	0.866	0.866	0.866	0.866	0.866	0.866	0.866
	CR	0.878	0.878	0.880	0.883	0.883	0.882	0.882	0.882
	AVE	0.784	0.785	0.789	0.793	0.794	0.792	0.792	0.792
Altruism	α	0.878	0.874	0.833	0.794	0.798	0.831	0.807	0.809
	CR	0.881	0.877	0.841	0.805	0.814	0.844	0.822	0.820
	AVE	0.711	0.705	0.640	0.583	0.597	0.646	0.610	0.605
Social and cultural norms	α	0.924	0.919	0.884	0.865	0.865	0.883	0.863	0.867
	CR	0.939	0.921	0.889	0.866	0.869	0.887	0.860	0.867
	AVE	0.838	0.796	0.728	0.683	0.688	0.724	0.672	0.685
Acceptance Intention	α	0.926	0.932	0.911	0.886	0.917	0.909	0.916	0.903
	CR	0.926	0.932	0.911	0.888	0.918	0.909	0.916	0.905
	AVE	0.862	0.873	0.837	0.798	0.849	0.834	0.845	0.826

Note: Test 1—Personal washing; Test 2—Clothes washing; Test 3—Pet washing; Test 4—Indoor plant watering; Test 5—Car washing; Test 6—Toilet flushing; Test 7—Outdoor green irrigation; Test 8—Road spraying.

**Table 2:** Structural equation model fit index.

Fit Indicator	Fit Standards	Test 1	Test 2	Test 3	Test 4	Test 5	Test 6	Test 7	Test 8
χ^2^/df	1 < χ^2^/df < 3	1.997	1.516	2.124	1.599	2.386	2.005	2.518	1.966
RMR	<0.05	0.034	0.027	0.030	0.020	0.024	0.021	0.022	0.021
RMSEA	<0.08	0.039	0.028	0.041	0.030	0.046	0.039	0.048	0.038
GFI	>0.90	0.967	0.974	0.965	0.973	0.960	0.967	0.958	0.967
AGFI	>0.90	0.949	0.960	0.945	0.958	0.938	0.949	0.935	0.949
NFI	>0.90	0.975	0.982	0.971	0.976	0.967	0.973	0.965	0.972
RFI	>0.90	0.966	0.974	0.960	0.967	0.954	0.963	0.951	0.962
IFI	>0.90	0.988	0.994	0.985	0.991	0.980	0.986	0.978	0.986
TLI	>0.90	0.983	0.991	0.979	0.987	0.973	0.981	0.970	0.981
CFI	>0.90	0.988	0.994	0.985	0.991	0.980	0.986	0.978	0.986
PGFI	>0.50	0.619	0.624	0.618	0.623	0.615	0.620	0.614	0.619
PNFI	>0.50	0.703	0.707	0.700	0.703	0.696	0.701	0.695	0.701
PCFI	>0.50	0.712	0.716	0.709	0.714	0.706	0.711	0.705	0.711

Note: Test 1—Personal washing; Test 2—Clothes washing; Test 3—Pet washing; Test 4—Indoor plant watering; Test 5—Car washing; Test 6—Toilet flushing; Test 7—Outdoor green irrigation; Test 8—Road spraying.

**Table 3:** Demographic profile of respondents.

	Category	Percentage (%)		Category	Percentage (%)
Gender	Man	38.12	Education	Primary school	0.45
	Woman	61.88		Middle school	3.59
Age	18–29 years old	33.03		High School	4.19
	30–39 years old	32.88		Undergraduate	74.74
	40–49 years old	23.02		Postgraduate	17.04
	50–59 years old	8.67	Administrative regions *	Xiaodian	42.15
	60 years and older	2.39		Yingze	24.96
Monthly income per household (RMB)	Below 2001	6.28		Wanbailin	11.96
	2001–4000	18.54		Xinghualing	9.72
	4001–6000	25.86		Jiancaoping	7.47
	6001–8000	18.09		Jinyuan	3.74
	8001–10,000	12.56	Years of residence in Taiyuan City	Less than 5 years	18.24
	Above 10,000	10.61		5–10 years	17.64
	Refuse to answer	8.07		11–15 years	12.11
				16–20 years	8.97
				More than 20 years	43.05

* Taiyuan has 6 large residential communities as listed. Xiaodian and Yingze are old communities and a lot larger in size of population.

**Table 4:** Standardized path coefficients for structural equation models.

	Test 1	Test 2	Test 3	Test 4	Test 5	Test 6	Test 7	Test 8
	Personal washing	Clothes washing	Pet washing	Indoor plant watering	Car washing	Toilet flushing	Outdoor green irrigation	Road spraying
H1 Valuation of stormwater →Acceptance intention	−0.035	−0.035	−0.037	−0.115 **	−0.043	−0.061	0.019	−0.047
H2 Negative emotions →Acceptance intention	−0.072	−0.077	−0.050	−0.083	−0.047	−0.065	−0.057	−0.113
H3 Perceived health risks →Acceptance intention	−0.032	−0.034	0.018	0.012	−0.035	−0.031	0.058	0.067
H4 Trust in government →Acceptance intention	0.041	0.062	0.027	0.097	0.058	0.067	0.014	0.022
H5 Altruism →Acceptance intention	0.289 ***	0.185 ***	0.222 ***	0.184 ***	0.247 ***	0.241 ***	0.182 ***	0.159 ***
H6 Social and cultural norms →Acceptance intention	0.613 ***	0.658 ***	0.668 ***	0.691 ***	0.595 ***	0.670 ***	0.679 ***	0.695 ***

Note: *** significant at *p* < 0.001; ** significant at *p* < 0.01.

## Data Availability

The data presented in this study are available on request from the corresponding author. The data are not publicly available due to privacy issues.
